# Supplementary material for: Fusarium graminearum Infection Strategy in Wheat Involves a Highly Conserved Genetic Program That Controls the Expression of a Core Effectome
Source: Int J Mol Sci. 2022 Feb 8;23(3):1914. doi: 10.3390/ijms23031914 (PMC8836836; doi:10.3390/ijms23031914)
Supplement: Supplementary file 1 [file ijms-23-01914-s001.zip › Supplementary_Files/SupplementaryFigures.pdf]

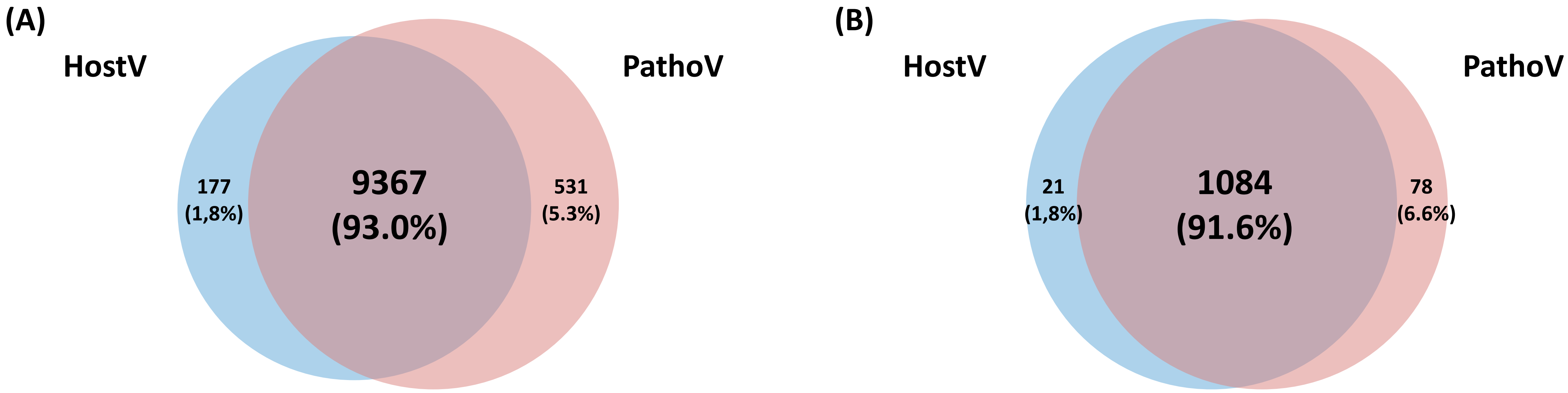

Figure S1: Number of detected transcripts in the HostV and PathoV experiments at the whole gene set (A) and at the secretome gene set (B) levels. Venn diagrams represent the number of genes expressed in the HostV (blue) and the PathoV (red) experiments. Genes were filtered per host and per strain with a 4CPM threshold in at least 3 samples.

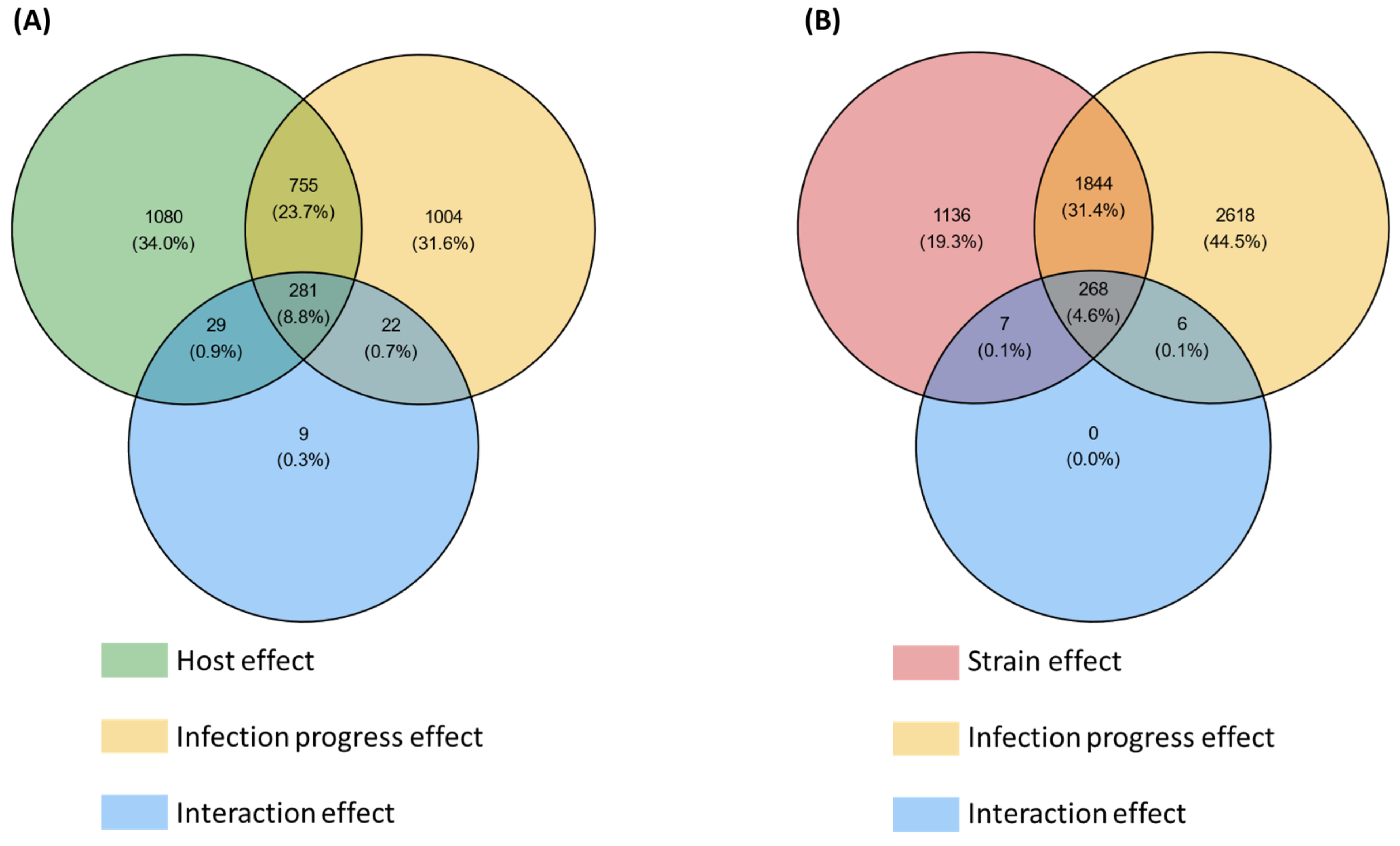

Figure S2: Number of genes from HostV (A) and PathoV (B) whole data sets significantly impacted by the different effects tested in the differential expression (DE) analysis. For each factor of the DE analysis, the Venn diagrams indicate the number of genes displaying significant expression variations. Significance threshold : p-value corrected by Benjamini-Hochberg method < 1e-3.

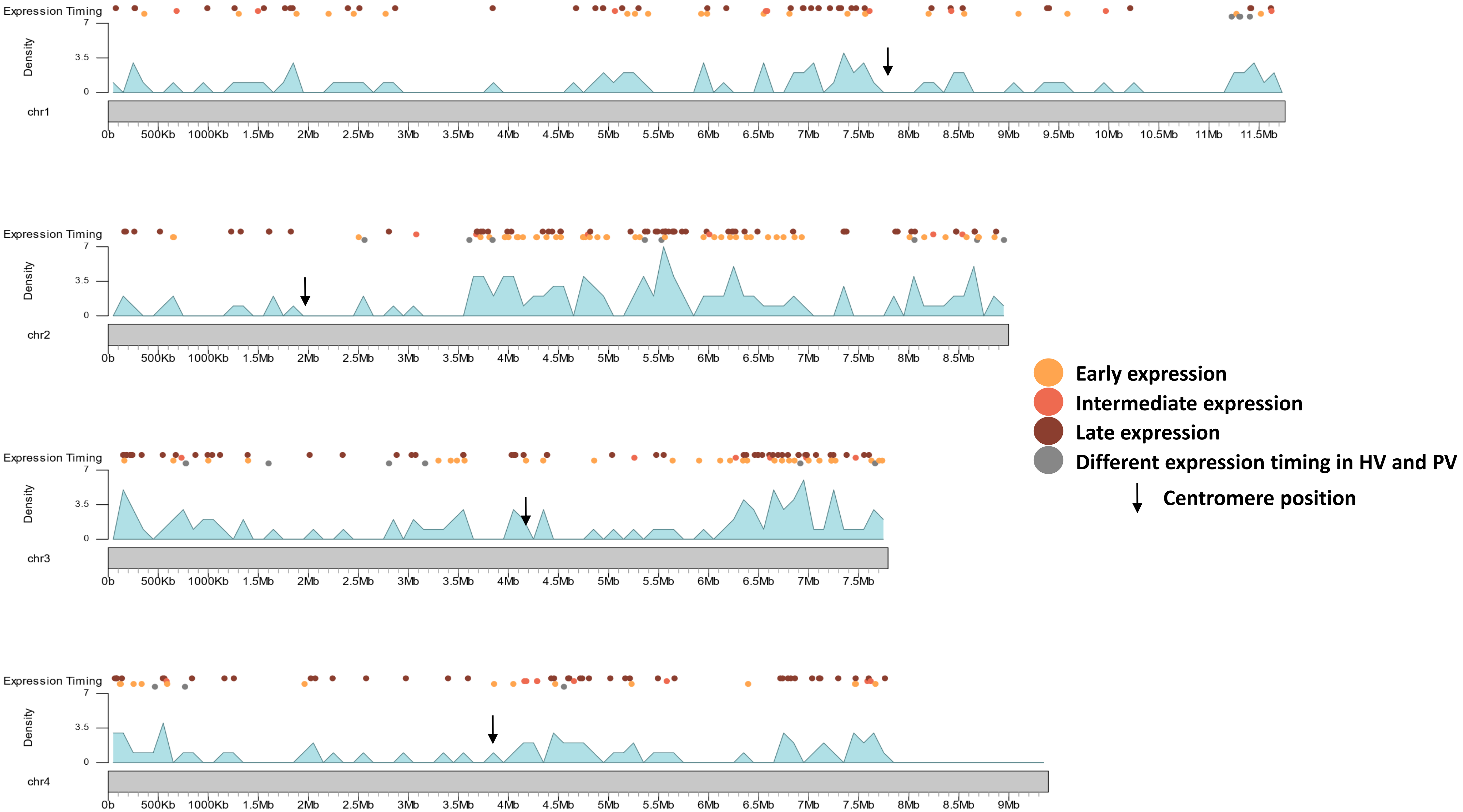

Figure S3: Genomic distribution of the core effectome genes according to their expression timing. Karyoplot represents the distribution density (light blue) of the genes on *Fusarium graminearum* chromosomes along with information about the expression timing. The window size for density representation is 100kB. Black vertical arrows indicate the centromere position.
